# Serum cytokine analysis in a cohort of advanced non-small cell lung cancer treated with PD-1 inhibitors reveals predictive markers of CXCL12

**DOI:** 10.3389/fimmu.2023.1194123

**Published:** 2023-06-09

**Authors:** Yanjun Xu, Ling Ding, Hui Li, Zhongsheng Peng, Kaibo Ding, Zhiyu Huang, Zichao Zhou, Mingying Xie, Junrong Yan, Sijie Feng, Yun Fan

**Affiliations:** ^1^ Department of Medical Thoracic Oncology, Zhejiang Cancer Hospital, Hangzhou Institute of Medicine (HIM), Chinese Academy of Sciences, Hangzhou, China; ^2^ Institute of Pharmacology and Toxicology, College of Pharmaceutical Sciences, Zhejiang University, Hangzhou, China; ^3^ Medical Department, Nanjing Geneseeq Technology Inc., Nanjing, Jiangsu, China; ^4^ School of Medicine, Henan Polytechnic University, Jiaozuo, Henan, China

**Keywords:** cytokine, CXCL12, immunotherapy, non-small cell lung cancer (NSCLC), programmed cell death ligand-1 (PD-L1)

## Abstract

**Background:**

The circulating predictive factors for the outcomes of advanced non-small cell lung cancer (NSCLC) patients receiving immune checkpoint inhibitors (ICIs) remain elusive. We aimed to assess the predictive value of circulating cytokines for outcomes.

**Methods:**

Serum samples of 102 advanced-stage NSCLC patients who underwent immunotherapy were collected at baseline. The relative levels of 37 cytokines were detected. PD-L1 expression was also analyzed.

**Results:**

Higher serum CXCL12 levels (top 33%) were a poor predictive biomarker for durable clinical benefit (DCB) (23.5% vs. 72.1%, *p*<0.001), progression-free survival (PFS) (3.76 vs. 14.40 months; *p*<0.001) and overall survival (OS) (12.20 vs. 44.84 months; *p*=0.008). Compared with PD-L1-negative patients, PD-L1-positive patients had a significantly higher objective response rate (ORR) (70.0% vs. 28.8%, *p*<0.001) and a prolonged mPFS (25.35 vs. 4.64 months, *p*=0.003) and tended to have an increased mOS (44.84 vs. 20.42 months, *p*=0.087). A signature comprising PD-L1<1% and the top 33% CXCL12 level was associated with the lowest ORR (27.3% vs. 73.7%, *p*<0.001) and DCB (27.3% vs. 73.7%, *p*<0.001) and the worst mPFS (2.44 vs. 25.35 months, *p*<0.001) and mOS (11.97 vs. 44.84 months, *p*=0.007). Area under the curve (AUC) analyses of PD-L1 expression, CXCL12 level and PD-L1 expression plus CXCL12 level to predict DCB or no durable benefit (NDB) showed AUC values of 0.680, 0.719 and 0.794, respectively.

**Conclusion:**

Our findings suggest that serum cytokine CXCL12 levels can predict the outcomes of patients with NSCLC receiving ICI. Moreover, the combination of CXCL12 levels and PD-L1 status can predict outcomes with a significantly improved discriminatory power.

## Background

Non-small cell lung cancer (NSCLC), including squamous and nonsquamous tumors, accounts for approximately 80% of all lung cancers. In advanced NSCLC, platinum-based chemotherapy is associated with a poor 5-year survival rate (4.5%) (1). Currently, therapeutic strategies for advanced NSCLC with no driver gene mutations have turned to immunotherapy, such as immune checkpoint inhibitors (ICIs) that target the programmed cell death-1 (PD-1)/programmed cell death ligand-1 (PD-L1) axis or CTLA-4. According to the National Comprehensive Cancer Network (NCCN) guidelines, the recommended first-line treatment for advanced wild-type NSCLC with PD-L1 expression levels ≥ 50% is the single agent pembrolizumab. For eligible patients with metastatic NSCLC, ICI plus chemotherapy is also recommended as first-line therapy regardless of the PD-L1 expression level. In fact, multiple trials have demonstrated that patients with advanced NSCLC who are eligible for ICI therapy survive longer, with 5-year survival rates ranging from 16% to 43%, depending on PD-L1 expression levels and the ICI line of treatment (2). Moreover, several ICIs, including pembrolizumab, nivolumab, and atezolizumab, have been approved as subsequent therapies for patients with advanced or metastatic NSCLC if they have not yet received a PD-1/PD-L1 inhibitor.

Nevertheless, only a minority of patients with advanced-stage NSCLC achieve long-term survival after ICI therapy (3). Thus, there is a need to improve patient selection and identify predictive biomarkers for durable clinical benefit (DCB) or no durable benefit (NDB). Biomarker studies have revealed some genomic signatures in tumor biopsies that are predictive of the efficacy and outcomes of ICIs, including positive PD-L1 expression, high tumor mutational burden (TMB), mismatch repair status, T-effect or interferon gamma gene signatures, and T-cell infiltration status (4–8). However, there are limitations associated with biomarker studies of tumor biopsies. First, a considerable proportion of patients with advanced NSCLC have difficulties providing enough tumor tissue for molecular testing. Second, tumor heterogeneity exists among different metastatic organs. Third, the variable definition of PD-L1 positivity and high TMB cutoff values in tumor biopsies cannot be ignored. Moreover, challenges in standardizing the testing platform have limited the clinical application of the biomarkers mentioned above.

The potential of circulating biomarkers to be used as predictive factors for DCB or NDB after ICI treatment in real-world clinical practice has attracted increased attention. Recent reports have indicated that the characteristics of the host immune system are critical in predicting the response to ICIs. For example, a lower neutrophil-to-lymphocyte ratio at baseline was found to be significantly associated with improved overall survival (OS) and progression-free survival (PFS) in patients treated with ICIs (9). Thus, we particularly focused on long- or short-term survivors of advanced NSCLC who underwent ICI treatment and investigated the role of cytokines in predicting PFS and OS. In this study, the serum samples and complete survival follow-up data of patients with advanced NSCLC who received immunotherapy in a real-world clinical practice were examined to identify circulating factors associated with survival.

## Methods

### Patients

A total of 1320 patients with NSCLC who received anti-PD-1 therapeutic agents at Zhejiang Cancer Hospital between January 2017 and December 2021 were retrospectively screened. Only patients with advanced NSCLC who had serum samples, tissue samples at baseline, and complete survival data were included. The major inclusion criteria were as follows: >18 years old; Eastern Cooperative Oncology Group performance status (ECOG-PS) score of 0-1; advanced NSCLC; treated with anti-PD-1 therapeutic agents; and radiologically evaluable according to Response Evaluation Criteria in Solid Tumors (RECIST) version 1.1. The major exclusion criteria were as follows: inadequate tissue samples for PD-L1 detection; harboring *EGFR* or *BRAF* mutations or *ALK/ROS1* rearrangements; and incomplete follow-up data. A total of 102 patients who met the inclusion criteria were enrolled. The following clinicopathological features were analyzed: age, sex, smoking history, brain metastasis, liver metastasis, line of treatment, and immune-related adverse events (irAEs). PFS was defined as the time from the initiation of immunotherapy to disease progression. OS was calculated from the initiation date of immunotherapy to death from any cause or the last follow-up date. Durable clinical benefit (DCB) was defined as confirmed absence of progressive disease for at least 6 months after ICI; whereas, cases showing progression of disease or stable disease lasting ≤6 months were considered as showing no durable benefit (NDB) (10). All patients were evaluated for tumor response, PFS, and OS. The follow-up rate was 100%, and the last follow-up date was July 12, 2022.

### Detection and analysis of circulating cytokines

The Proteome Profiler Human Cytokine Array Kit (ARY005B; R&D Systems, Minneapolis, MN) was designed to detect the relative levels of 37 cytokines, chemokines, and acute phase proteins in the serum (**Table S1**). Serum samples were collected and subjected to a cytokine array according to the manufacturer’s instructions (11–13). In brief, blood samples were clotted for 30 min at room temperature and centrifuged for 15 min at 1000 × *g*, after which the sera were transferred to new tubes. Array membranes previously spotted with capture antibodies by the manufacturer were incubated with 200 μL of serum overnight at 4°C. After washing, the membranes were further incubated with a streptavidin-HRP antibody (#893019) for 30 min at room temperature and developed using 2.5 mL of Chemi-Reagent Mix (#894287, #894288). The immunoblots were imaged using a ChemiDoc Imaging System (Amersham Imager 680; Amersham, Little Chalfont, UK), after which cytokine spots were normalized to the reference and semiquantitated using ImageJ software (NIH, Bethesda, MD). All cytokine arrays were stained in parallel to avoid different exposure times.

The Human Cytokine Array Kit is a rapid, sensitive, and economic tool to simultaneously detect cytokine differences between samples. 37 human cytokines capture antibodies selected by manufacturer have been spotted in duplicate on nitrocellulose membranes. Serum is mixed with a cocktail of biotinylated detection antibodies. The serum and antibody mixture is then incubated with the Human Cytokine Array membrane. Any cytokine/detection antibody complex present is bound by its cognate immobilized capture antibody on the membrane. Following a wash to remove unbound material, Streptavidin-HRP and chemiluminescent detection reagents are added sequentially. Light is produced at each spot in proportion to the amount of cytokine bound.

### Quantification and normalization the circulating cytokines signals

First of all, Image J software was used to perform gray analysis on the exposed membrane, and the mean gray value was used to quantify the signal of each cytokine. Besides, the region without signal was selected as the background signal, and the gray value of background signal was subtracted as the final gray value of each cytokine. About how to quantified the signal of each sample, the manufacturer designed three pairs of reference spots on each membrane, and the signal of reference spots on each membrane would reflect the same amount of human cytokines. In order to compare the signal difference between different samples, we quantified by using the ratio of each factor to the mean of the six standard wells to compare the cytokine differences between patients. The higher the ratio, the higher the expression level of cytokine in patients.

### Immunohistochemistry for PD-L1 expression

Formalin-fixed and paraffin-embedded (FFPE) tissues were used for immunohistochemistry to determine PD-L1 levels by two experienced pathologists, using an anti-human PD-L1 antibody (22C3 pharmDx kit; Dako, Carpinteria, CA) according to the manufacturer’s recommendations. The PD-L1 expression level was evaluated by tumor proportion score (TPS) and TPS ≥1% was identified as positive. PD-L1 TPS ≥ 50% was defined as strong PD-L1 positivity.

### Statistical analysis

Quantitative data are displayed as the median (range) or number of patients (percentage). Comparisons of proportions between groups were performed using Fisher’s exact test. For survival analyses, Kaplan-Meier curves were compared using the log-rank test, and hazard ratio (HR) and 95% confidence interval (CI) were calculated using the Cox proportional hazards model. Univariate and multivariate analyses were performed to study the associations between different variables and PFS and OS. Two-sided P values < 0.05 were considered significant for all tests unless indicated otherwise. All statistical analyses were performed using R (v.3.6.0).

## Results

### Baseline clinicopathological characteristics and their correlation with ICI benefit

A total of 1320 patients were diagnosed with NSCLC and treated with immunotherapy between January 2017 and December 2021 at Zhejiang Cancer Hospital. Patients who carried driver gene mutations (*EGFR* or *BRAF* mutations or *ALK/ROS1* rearrangements), who received ICIs as neoadjuvant treatment or less than two cycles of ICIs, who were lost to follow-up, and who did not complete the tumor response assessment were excluded from the study. Of the 1320 patients, 102 (7.7%) with advanced NSCLC who had serum samples and matched tissue samples at baseline were included in the analysis. The median age of the patients was 61 years (range: 43-83 years). The predominant histology of the tumors was squamous cell carcinoma (57/102, 55.9%). A total of 83 patients (83/102, 81.4%) had a smoking history. Nine (8.8%) patients presented with baseline brain metastasis at the initiation of ICI treatment, and 14 (13.7%) patients had baseline liver metastasis. ICIs were used as first-line treatment for 56 (54.9%) patients and as second-line treatment for 46 (45.1%) patients. A total of 47 (46.1%) patients were treated with ICIs as monotherapy. The patient characteristics are listed in **Table 1**.

**Table 1:** Patient demographics and baseline clinicopathological characteristics.

Characteristic	Cohort N = 102 (%)
Age	
Median (y, range)	61 (43-83)
< 65 y	66 (64.7%)
≥ 65 y	36 (35.3%)
Sex	
Male	88 (86.3%)
Female	14 (13.7%)
Smoking	
Yes	83 (81.4%)
No	19 (18.6%)
Pathology	
Adenocarcinoma	45 (44.1%)
Squamous cell carcinoma	57 (55.9%)
PD-L1 TPS	
< 1%	52 (51.0%)
1-49%	37 (36.3%)
≥ 50%	13 (12.7%)
Brain metastasis	
Yes	9 (8.8%)
No	93 (91.2%)
Liver metastasis	
Yes	14 (13.7%)
No	88 (86.3%)
Line of treatment	
1	56 (54.9%)
2	46 (45.1%)
Treatment strategy	
Monotherapy	47 (46.1%)
Combined chemotherapy	55 (53.9%)

In our cohort, the estimated median PFS (mPFS) and OS (mOS) were 10.49 months (95% CI: 5.95-16.60) and 36.36 months (95% CI: 21.07-Not reached), respectively. The overall objective response rate (ORR) was 49.0%. 57 (55.9%) patients achieved DCB, with a PFS of ≥ 6 months (**Table S2**). The baseline clinicopathological characteristics and irAEs were also analyzed to predict PFS or OS after ICI treatment. Using univariate analysis, irAEs was found to be a significantly predictive factor for PFS (Yes vs. No, HR=0.44, 95% CI [0.25-0.76], *p*=0.003) and OS (Yes vs. No, HR=0.37, 95% CI [0.15-0.87], *p*=0.018) (**Table S3**). Besides, patients with liver metastasis at baseline showed significantly worse OS (Yes vs. No, HR=2.52, 95% CI [1.20-5.31], *p*=0.012) and trended towards worse PFS (Yes vs. No, HR=1.71, 95% CI [0.90-3.27], *p*=0.098) (**Table S3**).

### Identification of a circulating cytokine signature and its correlation with ICI benefit

**Figure 1:**
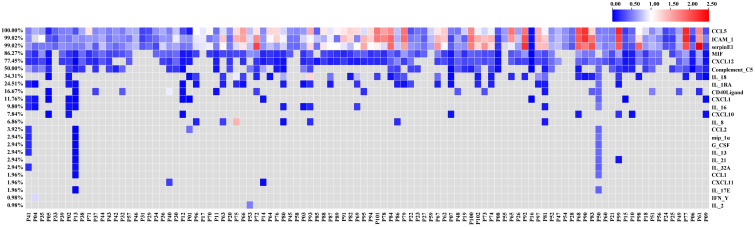
Heatmap of the relative levels of circulating cytokines analyzed in this study. The heatmap shows only the cytokines detected in at least one patient.

Firstly, we assessed the serum cytokine levels of 102 patients with advanced NSCLC before ICI treatment. The 37 cytokines studied in this study were listed in **Table S1**. The heatmap of the relative levels of circulating cytokines detected in at least one patient was shown in **Figure 1**. The analysis results included only cytokines with positive (upregulated) expression in ≥ 34 (1/3) patients, including CCL5, ICAM-1, serpinE1, MIF, CXCL12, complement C5, and IL-18 (**Table 2**). When the high- or low-cytokine groups were defined with cutoffs of dichotomy, no association was found between cytokine expression and PFS or OS (data not shown). We then defined the high-cytokine group as patients with a cytokine value ≥ the top 33% (1/3) and the low-cytokine group as patients with a value < the bottom 67% (2/3).

**Table 2:** Univariate Cox analysis of PFS or OS by the levels of circulating cytokines in NSCLC patients who received immunotherapy.

Cytokine	PFS		OS	
	HR (95% CI)	P value	HR (95% CI)	P value
CCL5				
Top 1/3 vs. Bottom 2/3	1.24 (0.73-2.10)	0.434	0.78 (0.32-1.92)	0.585
ICAM-1				
Top 1/3 vs. Bottom 2/3	1.57 (0.94-2.62)	0.086	0.8 (0.33-1.96)	0.625
serpinE1				
Top 1/3 vs. Bottom 2/3	1.46 (0.86-2.50)	0.160	0.95 (0.38-2.36)	0.913
MIF				
Top 1/3 vs. Bottom 2/3	1.62 (1.00-2.61)	**0.046**	1.03 (0.53-2.00)	0.934
CXCL12				
Top 1/3 vs. Bottom 2/3	2.50 (1.55-4.04)	**1e-04**	2.31(1.22-4.36)	**0.008**
Complement C5				
Top 1/3 vs. Bottom 2/3	0.94 (0.57-1.55)	0.807	1.22 (0.65-2.30)	0.537
IL-18				
Top 1/3 vs. Bottom 2/3	1.31 (0.79-2.18)	0.292	0.946 (0.43-2.08)	0.890

The bold values means *p* < 0.05.

**Figure 2:**
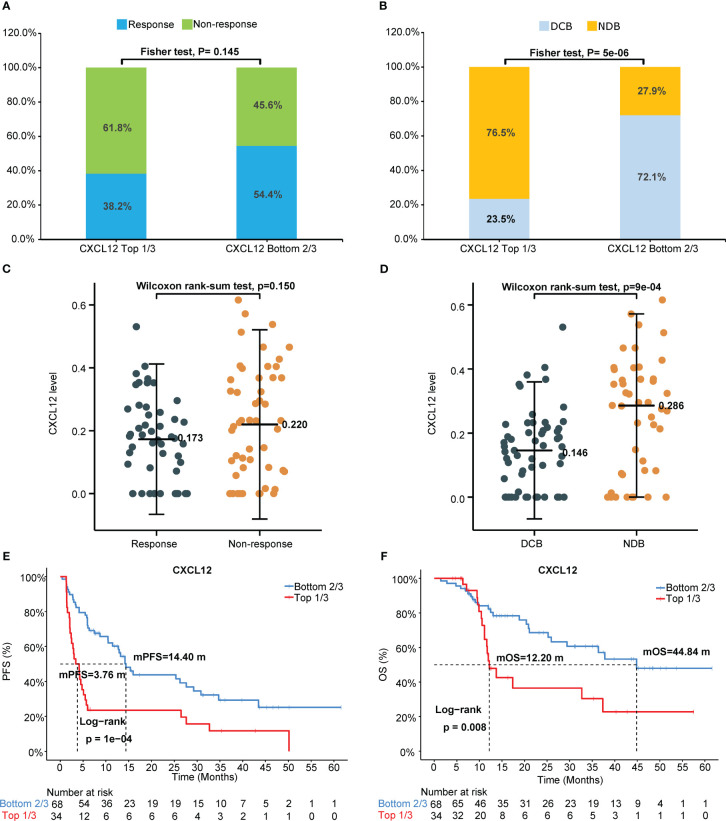
Pretreatment circulating chemokine CXCL12 levels predict outcomes of immunotherapy. **(A)** Response or nonresponse and **(B)** DCB or NDB of the patient proportions according to the levels of circulating CXCL12, stratified by the cutoffs of the top 33% (1/3) and bottom 67% (2/3). Pretreatment circulating CXCL12 levels in **(C)** patients who responded or did not respond to immunotherapy and in **(D)** patients with DCB or without DCB (NDB). Probability of **(E)** PFS or **(F)** OS according to circulating CXCL12 level, stratified by the cutoffs of the top 33% and bottom 67%. DCB, durable clinical benefit. NDB, no durable benefit.

Univariate Cox analysis of PFS or OS by the levels of circulating cytokines in NSCLC patients who received immunotherapy showed that pretreatment circulating cytokine CXCL12 levels predicted the outcomes of immunotherapy (**Table 2**). Representative images of the arrays (CXCL12 high and low) was shown in **Figure S1A, B**. The response rates of the patient proportions according to the levels of circulating CXCL12, stratified by the cutoffs of the top 33% (1/3) and bottom 67% (2/3), were 38.2% and 54.4%, respectively (*p*=0.015, **Figure 2A**). The DCB rates of the patient proportions according to the levels of circulating CXCL12, stratified by the cutoffs of the top 33% (1/3) and bottom 67% (2/3), were 23.5% and 72.1%, respectively (*p*<0.001, **Figure 2B**). The pretreatment circulating CXCL12 level in patients who responded to immunotherapy was lower than that in patients who did not respond to immunotherapy (*p*=0.15, **Figure 2C**). The pretreatment circulating CXCL12 level in patients with DCB was significantly lower than that in patients with NDB (*p*<0.001, **Figure 2D**). The results also showed that CXCL12, which was in the top 33% (1/3), was a poor predictive factor for PFS (mPFS 3.76 vs. 14.4 months; *p*<0.00) and OS (mOS 12.20 vs. 44.84 months; *p*=0.008) (**Table 2**, **Figures 2E, F**). The associations of pretreatment CXCL12 levels with PFS (4.14 vs. 14.20 months, *p*=0.002, **Figure S2A**) and OS (11.97 months vs. Not reached, *p*=0.017, **Figure S2B**) in patients who received chemo-immunotherapy were also shown in the Supplemental data. No significant association was found between pretreatment CXCL12 levels and PFS or OS in patients who received mono-immunotherapy (**Table S4**).

In addition, we found that the level of MIF was a poor predictive factor for PFS. The association of pretreatment circulating MIF levels with immunotherapy efficacy in NSCLC was shown in **Figure S3**. No significant differences were found between response or nonresponse and DCB or NDB of patient proportions according to the baseline levels of circulating MIF (**Figures S3A, B**). MIF in the top 33% (1/3) was a poor predictive factor for PFS (mPFS 5.65 vs. 14.2 months; *p*=0.046) but not for OS (mOS 32.71 vs. 37.81 months; *p*=0.93) (**Table 2**, **Figures S3C, D**). No significant relationships were observed for the other cytokines (**Table 2**).

We further do the multivariate Cox analysis of PFS or OS by clinical parameters, PD-L1 status and CXCL12 level. The results also showed that CXCL12, which was in the top 33% (1/3), was a poor predictive factor for PFS (HR=3.01, 95% CI [1.63-5.55], *p*<0.00) and OS (HR=4.63, 95% CI [1.81-11.80], *p*=0.001) (**Table 3**).

**Table 3:** Multivariate Cox analysis of PFS or OS by clinical parameters, PD-L1 status and CXCL12 level.

Factor	PFS		OS	
	p value	HR (95% CI)	p value	HR (95% CI)
PD_L1 TPS ≥1% vs. <1%	**0.003**	0.48 (0.29-0.78)	0.247	0.68 (0.36-1.30)
Liver Metastasis Yes vs. No	**0.025**	2.17 (1.10-4.29)	**0.043**	2.24 (1.03-4.90)
irAEs Yes vs. No	**0.001**	0.39 (0.22-0.69)	**0.046**	0.41 (0.17-0.98)
CXCL12 Top 1/3 vs. Bottom 2/3	**4e-04**	3.01 (1.63-5.55)	**0.001**	4.63 (1.81-11.80)
MIF Top 1/3 vs. Bottom 2/3	0.915	1.03 (0.56-1.90)	0.079	0.42 (0.16-1.10)

irAE, immune-related adverse events; TPS, tumor proportion score; CI, confidence interval; HR, hazard ratio; the bold values, *p* < 0.

### Relationship between PD-L1 expression and ICI benefit

**Figure 3:**
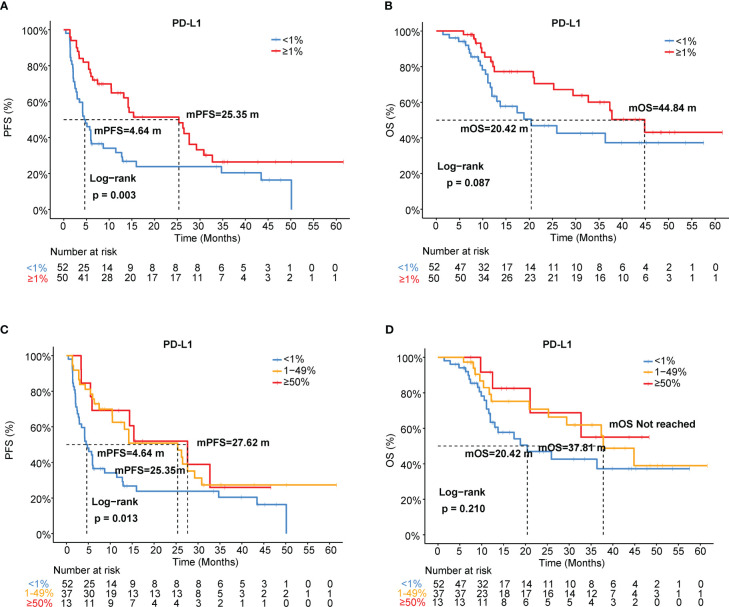
Association of PD-L1 expression with immunotherapy efficacy in NSCLC. **(A)** PFS and **(B)** OS curves for patients with PD-L1 TPS≥1% and <1%. **(C)** PFS and **(D)** OS curves for patients with PD-L1 TPS<1%, 1-49%, and ≥50%. TPS, tumor proportion score.

Using a cutoff of TPS ≥ 1% (22C3 antibody), PD-L1 positivity was observed in 49.0% (50/102) of patients, and PD-L1 expression ≥ 50% was detected in 13 (12.7%) patients (**Table 1**). Representative IHC images with different TPS of PD-L1 was showed in the **Figure S1C**. Using univariate and multivariable analyses, PD-L1 was found to be an independent predictive factor for PFS (HR=0.5, 95% CI [0.31-0.80], *p*=0.003; HR=0.48, 95% CI [0.29-0.78], *p*=0.003) but not for OS (HR=0.58, 95% CI [0.31-1.09], *p*=0.087; HR=0.68, 95% CI [0.36-1.30], *p*=0.247) in our cohort (**Figures 3A, B**, **Table 3**). Patients with PD-L1 TPS≥ 1% showed a significantly prolonged mPFS (25.35 vs. 4.64 months, *p*=0.003) and a trend toward increased mOS (44.84 vs. 20.42 months, *p*=0.087) compared with those with negative PD-L1 expression in the whole population (**Figures 3A, B**). Moreover, the associations of the pretreatment PD-L1 expression status with PFS and OS in patients who received mono-immunotherapy or chemo-immunotherapy were also shown in **Table S4**. In the mono-immunotherapy cohort, patients with PD-L1 TPS≥1% showed longer mPFS (25.35 vs. 3.80 months, *p*=0.017, **Figure S4A**) and OS (44.84 vs. 15.57 months, *p*=0.03, **Figure S4B**) compared to that with PD-L1 TPS< 1%. However, no significant association was found between the pretreatment PD-L1 expression status and PFS or OS in patients who received chemo-immunotherapy (**Table S4**). The mPFS were 4.64, 25.35 and 27.62 months for patients with PD-L1<1%, 1-49%, and ≥50%, respectively (**Figure 3C**, **Table S2**). The mOS were 20.42, 37.81 months and not reached for patients with PD-L1 <1%, 1-49%, and ≥50%, respectively (**Figure 3D**, **Table S2**).

In addition, we investigated the association between different levels of PD-L1 expression and ORR or DCB. The response/non-response proportions of patients stratified by PD-L1 status (TPS ≥1% and <1%) were 70.0% and 28.8%, respectively (*p*<0.001, **Figure S5A**). The DCB rates of the proportions of patients stratified by PD-L1 status (TPS ≥1% and <1%) were 73.7% and 38.5%, respectively (*p*<0.001, **Figure S5B**). The ORRs were 28.8%, 70.3%, and 69.2% in patients with PD-L1<1%, 1–49%, and ≥50%, respectively (**Table S2**, **Figure S5C**). The DCB rates of the patient proportions according to PD-L1 expression levels (TPS<1%, 1-49%, and ≥50%) were 38.5%, 73.7%, and 69.2%, respectively (*p*=0.001, **Figure S5D**).

### Clinical outcomes based on the baseline cytokine signature and PD-L1 status

**Figure 4:**
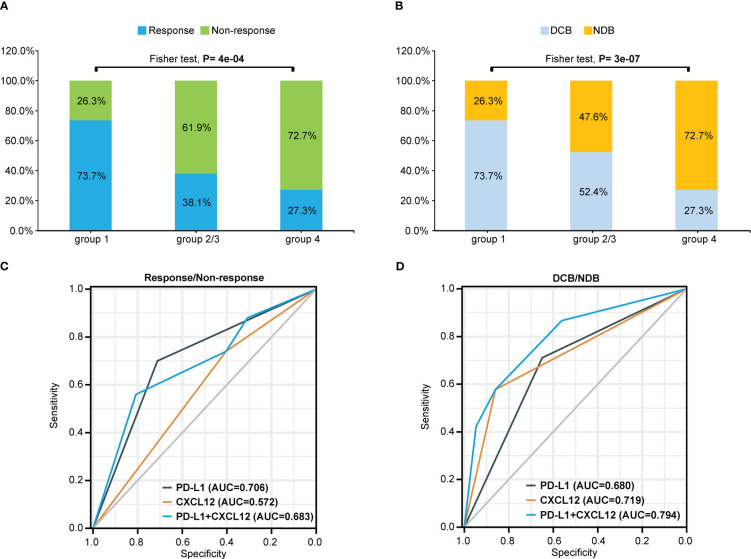
Combination of pretreatment PD-L1 expression and circulating chemokine CXCL12 levels improved the prediction capability of immunotherapy outcomes. **(A)** Response or nonresponse and **(B)** DCB or NDB of the patient proportions in different groups stratified by the PD-L1 TPS plus CXCL12 level at baseline. The patients were divided into four groups: group 1 (TPS≥1% and CXCL12 bottom 67%); group 2 (TPS<1% and CXCL12 bottom 67%); group 3 (PD-L1≥1% and CXCL12 top 33%); and group 4 (PD-L1<1% and CXCL12 top 33%). Groups 2 and 3 were pooled together. AUC analyses of PD-L1 expression, CXCL12 level and PD-L1 expression plus CXCL12 level to predict **(C)** response/nonresponse or **(D)** DCB/NDB. TPS, tumor proportion score; AUC, area under the curve; DCB, durable clinical benefit. NDB, no durable benefit.

**Figure 5:**
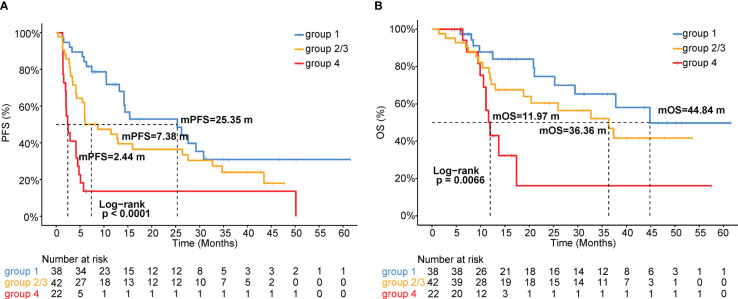
Kaplan-Meier analysis of **(A)** PFS or **(B)** OS stratified by the combination of pretreatment PD-L1 expression and circulating CXCL12 levels. Groups 2 and 3 were pooled together.

As shown in **Figures 2** and **3**, patients with negative PD-L1 (<1%) expression tended to have decreased mPFS (*p*<0.05) and mOS (*p*>0.05), and those with CXCL12 levels in the top 33% (1/3) had poor mPFS (*p*=1e-04) and mOS (*p*=0.008). Thus, when we combined PD-L1 expression with CXCL12 levels, the discriminatory power was significantly improved (**Figures 4**, **5**). The 102 patients were then separated into four groups as follows: group 1, PD-L1 TPS ≥1% and CXCL12 bottom 67% (2/3); group 2, PD-L1 TPS <1% and CXCL12 bottom 67% (2/3); group 3, PD-L1 TPS ≥1% and CXCL12 top 33% (1/3); and group 4, PD-L1 TPS <1% and CXCL12 top 33% (1/3). Groups 2 and 3 were pooled together. The data showed that the combination of pretreatment PD-L1 expression and circulating cytokine CXCL12 levels improved the prediction capability of immunotherapy outcomes. The response rates of the patient proportions in group 1, group 2/3, and group 4 stratified by the PD-L1 TPS plus CXCL12 level at baseline were 73.7%, 38.1% and 27.3%, respectively (*p*<0.001, **Figure 4A**). The DCB rates of the patient proportions in group 1, group 2/3, and group 4 were 73.7%, 52.4% and 27.3%, respectively (*p*<0.001, **Figure 4B**). Area under the curve (AUC) analyses of PD-L1 expression, CXCL12 level and PD-L1 expression plus CXCL12 level in predicting response/nonresponse showed AUC values of 0.706, 0.572 and 0.683, respectively (**Figure 4C**). AUC analyses of PD-L1 expression, CXCL12 level and PD-L1 expression plus CXCL12 level in predicting DCB/NDB showed AUC values of 0.680, 0.719 and 0.794, respectively (**Figure 4D**). Group 4 showed the worst mPFS (2.44 vs. 7.38 vs. 25.35 months; *p*<0.001) and mOS (11.97 vs. 36.36 vs. 44.84 months; *p*=0.007) compared with group 2/3 and group 1 (**Figure 5**).

The response or nonresponse and DCB or NDB of the patient proportions and Kaplan-Meier analysis of PFS or OS in the four groups stratified by the PD-L1 TPS plus CXCL12 level at baseline are also shown in **Figure S6**.

## Discussion

Although PD-L1 is a good predictive biomarker for DCB, it is not unique, and there is still an urgent need to identify additional biomarkers to predict the survival of patients receiving ICI treatment. In this study, we mainly assessed the predictive value of circulating cytokine levels in Chinese patients with advanced NSCLC following ICI treatment and found that the circulating cytokine CXCL12 level may help predict DCB, PFS, and OS. More interestingly, when we combined PD-L1 expression with CXCL12 levels, the discriminatory power was significantly improved. In this scenario, when we obtained the results of PD-L1 expression, it is important and valuable to analyze the expression level of CXCL12 in the serum, which was easier to collect from patients. Because the level of CXCL12 expression could further identify the patients who would potentially benefit from immunotherapy, regardless of whether they are PD-L1 negative or positive.

In our cohort, the patients’ clinical characteristics were also analyzed, and the results indicated that liver metastasis at baseline was a poor prognostic factor for survival benefit, which was consistent with previous reports (14, 15). In our study, irAEs appeared to be associated with an improved benefit of immunotherapy, which was also consistent with other studies’ findings (16–18). The above results also show that our cohort, including advanced NSCLC patients who underwent immunotherapy in the real world, is very representative.

The role of PD-L1 as a biomarker of efficacy and long-term survival in advanced NSCLC has been discussed in many studies (4, 5, 19). In our cohort, using a cutoff of TPS ≥1%, PD-L1 positivity was observed in 49.0% of patients, and PD-L1 expression ≥50% was detected in 12.7% of patients. Compared with PD-L1-negative patients, PD-L1-positive patients had a significantly higher ORR and DCB rate and showed longer mPFS, but the trend of increased mOS was not statistically significant. In line with our results, two meta-analyses indicated that the HRs for the ORR were 2.18 (95% CI 1.45-3.29; *p*=0.0002) and 2.08 (95% CI 1.49-2.91; *p*<0.01) in patients whose tumors had PD-L1 expression >1% and in those whose tumors had PD-L1 expression <1% (20, 21). The PD-L1 status in cancer cells is not always sufficient for identifying patients who are more likely to respond to ICI treatment, especially in patients who receive chemoimmunotherapy or dual immunotherapy combination (22–24). Data from Keynote-189, Orient-11 and CheckMate-9LA showed that PFS and OS benefits could be achieved with chemoimmunotherapy or dual immunotherapy combination, regardless of PD-L1 TPS expression <1%, 1-49%, or ≥50% (22–24). Data from Rationale 307 showed that a PFS benefit could be derived from chemoimmunotherapy, regardless of PD-L1 TC expression (1%, 25%, or 50%), and OS data were not disclosed (25). Consistent with these studies, we found that the pretreatment PD-L1 expression status was significantly associated with PFS (3.80 vs. 25.35 months, *p*=0.017) and OS (15.57 vs. 44.84 months, *p*=0.03) in patients who received monoimmunotherapy but not in patients who received chemoimmunotherapy.

Recently, investigators have focused on other potential biomarkers, such as T-cell infiltration, molecular driver genes, CD8+ T-cell expression, or cytokines (8, 26, 27). To reduce the impact of tumor cell heterogeneity, we determined the serum cytokine levels at baseline in our cohort. Interestingly, when defining the high- and low-cytokine groups based on cutoffs with trichotomy, CXCL12 in the top 33% (1/3) was a poor prognostic factor for DCB, PFS and OS. Given that combinations of biomarkers are likely needed due to the multifactorial nature of cancer–immune interactions, we further combined PD-L1 status with the CXCL12 level in an attempt to distinguish DCB and NDB patients. AUC analyses of PD-L1 expression, CXCL12 level and PD-L1 expression plus CXCL12 level to predict DCB/NDB showed AUC values of 0.680, 0.719 and 0.794, respectively. Subsequently, group 4 patients (PD-L1<1% and CXCL12 top 33% (1/3) were found to have the lowest ORR and DCB rate and the worst mPFS and mOS. In contrast, group 1 patients with PD-L1≥1% and CXCL12 bottom 67% (2/3) were found to have the highest ORR and DCB rate and the best mPFS and mOS. Our findings suggest that the combination of circulating cytokine CXCL12 levels and PD-L1 status can predict the survival of advanced NSCLC patients treated with ICIs, regardless of monotherapy or chemoimmunotherapy.

CXCL12, the ligand for CXCR4, is a member of the chemokine/chemokine receptor system that regulates the trafficking of immune cells (28–30). Cancer cells interact with the chemokine/chemokine receptor system to escape immune attack partially by coating themselves with CXCL12. Fearon et al. further found that cancer cells could restrict T-cell motility and suppress the intratumoral accumulation of T cells by assembling a transglutaminase-2 (TGM2)-dependent filamentous coating of CXCL12-keratin-19 (KRT19) heterodimers (31). Recent study also showed that loss of lymphatic-specific CXCL12 boosts T cell retention and enhances tumor control (32). These results also support our findings at a mechanistic level and explain why higher CXCL12 expression levels reduce the efficacy of immunotherapy.

Cytokines, including CXCL12, are key elements that coordinate the tumor microenvironment and control the tumor-immune cell interactions, and the intensive study of cytokines in tumor biology contributes to a better understanding of their mechanism of action and contribute to the development of new therapeutic strategies.

## Conclusions

In conclusion, our results suggest that serum CXCL12 levels are a potential biomarker for predicting the benefit and survival of patients with advanced NSCLC treated with ICIs. PD-L1 status has been considered a good predictive biomarker for ICI benefit, its limitations cannot be ignored. Indeed, our findings suggest that the combination of circulating cytokine CXCL12 levels and PD-L1 status can predict outcomes with a significantly improved discriminatory power. Given the ease of access to peripheral blood versus tumor tissue, the circulating cytokine CXCL12 may be considered the first test in the clinical setting to assess biomarkers. The limitations of our study included its small sample size and retrospective nature. Moreover, it was conducted at a single institution. These results warrant consideration of the predictive value of cytokines identified in our study and are worth further exploration in prospective clinical trials with a larger sample size for validation. The clinical potential of the methods described here also needs to be confirmed in the future.

## Data availability statement

The raw data supporting the conclusions of this article will be made available by the authors, without undue reservation.

## Ethics statement

The studies involving human participants were reviewed and approved by Zhejiang Cancer Hospital. The patients/participants provided their written informed consent to participate in this study.

## Author contributions

Conception and design: YX, LD, and YF; Administrative support: ZH and YF; Provision of study materials or patients: YX and ZH; Collection and assembly of data: YX, HL, MX, and ZZ; Data analysis and interpretation: YX, LD, SF, and JY; Manuscript writing: all authors. All authors contributed to the article and approved the submitted version.

## Supplementary material

Supplementary Figure 1 The representative images of the arrays and PD-L1. A. Representative patients with high expression of CXCL12. B. Representative patients with low expression of CXCL12. C. Representative IHC images with different TPS of PD-L1.

Supplementary Figure 2 The associations of pretreatment CXCL12 levels with PFS and OS in patients received chemo-immunotherapy. Kaplan-Meier curves of **(A)** PFS and **(B)** OS according to CXCL12 levels at baseline.

Supplementary Figure 3 The association of pretreatment circulating MIF with immunotherapy efficacy in NSCLC. **(A)** Response or nonresponse and **(B)** DCB or NDB of the patient proportions according to the baseline levels of circulating MIF, stratified by the cutoffs of the top 33% and bottom 67%. Kaplan-Meier curves of **(C)** PFS or **(D)** OS according to the circulating MIF level at baseline. DCB, durable clinical benefit. NDB, no durable benefit.

Supplementary Figure 4 The association of PD-L1 status with survival in patients who received mono-immunotherapy. Kaplan-Meier curves of **(A)** PFS and **(B)** OS according to the pretreatment PD-L1 expression status.

Supplementary Figure 5 The association of PD-L1 expression with immunotherapy response. **(A)** Response or nonresponse and **(B)** DCB or NDB of the patient proportions according to the PD-L1 expression levels (TPS≥1% and <1%). **(C)** Response or nonresponse and **(D)** DCB or NDB of the patient proportions stratified by PD-L1 status (TPS<1%, 1-49%, and ≥50%). TPS, tumor proportion score; DCB, durable clinical benefit; NDB, no durable benefit.

Supplementary Figure 6 Pretreatment PD-L1 expression plus circulating CXCL12 levels improve the prediction performance of immunotherapy outcomes. **(A)** Response or nonresponse and **(B)** DCB or NDB of the patient proportions in four groups stratified by the PD-L1 TPS plus CXCL12 level at baseline. Kaplan-Meier analysis of **(C)** PFS or **(D)** OS stratified by the PD-L1 expression plus CXCL12 level at baseline. The patients were divided into four groups: group 1 (TPS≥1% and CXCL12 bottom 67%); group 2 (TPS<1% and CXCL12 bottom 67%); group 3 (PD-L1≥1% and CXCL12 top 33%); and group 4 (PD-L1 < 1% and CXCL12 top 33%). TPS, tumor proportion score; DCB, durable clinical benefit; NDB, no durable benefit.
