# Supplementary figures and images for: Serum cytokine analysis in a cohort of advanced non-small cell lung cancer treated with PD-1 inhibitors reveals predictive markers of CXCL12
Source: Front Immunol. 2023 Jun 9;14:1194123. doi: 10.3389/fimmu.2023.1194123 (PMC10288851; doi:10.3389/fimmu.2023.1194123)

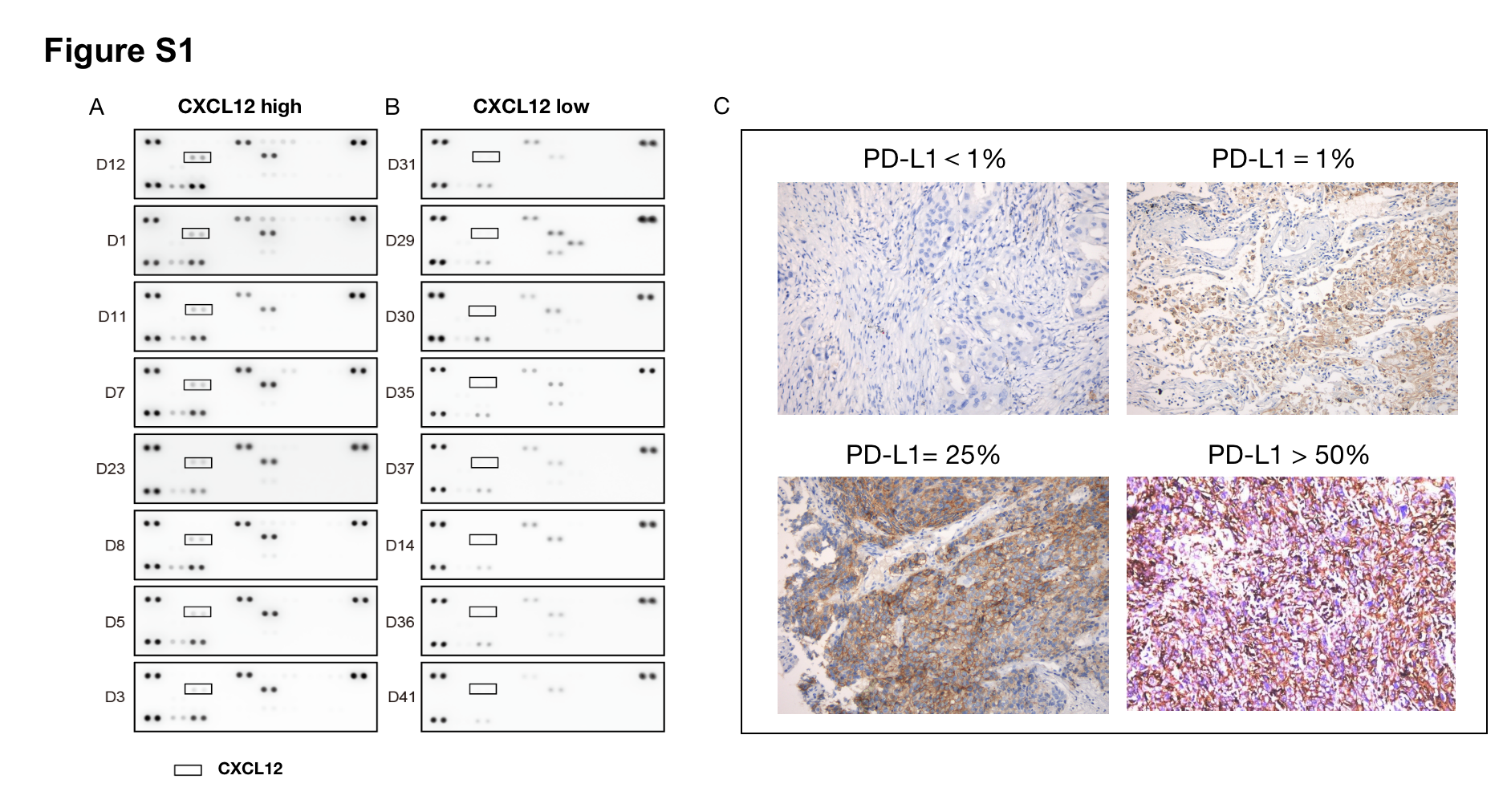

Supplement: Supplementary Figure 1 — The representative images of the arrays and PD-L1. A. Representative patients with high expression of CXCL12. B. Representative patients with low expression of CXCL12. C. Representative IHC images with different TPS of PD-L1. [file Image_1.tif]

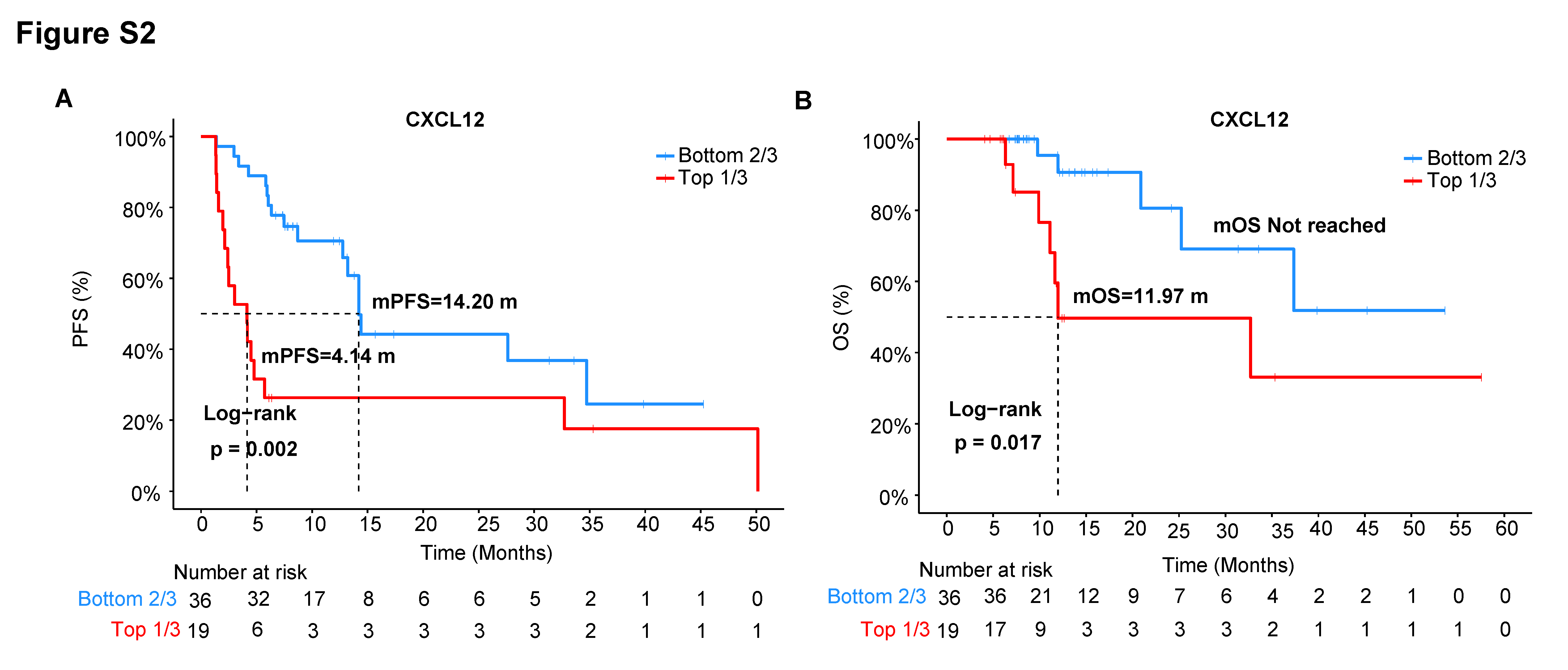

Supplement: Supplementary Figure 2 — The associations of pretreatment CXCL12 levels with PFS and OS in patients received chemo-immunotherapy. Kaplan-Meier curves of (A) PFS and (B) OS according to CXCL12 levels at baseline. [file Image_2.tiff]

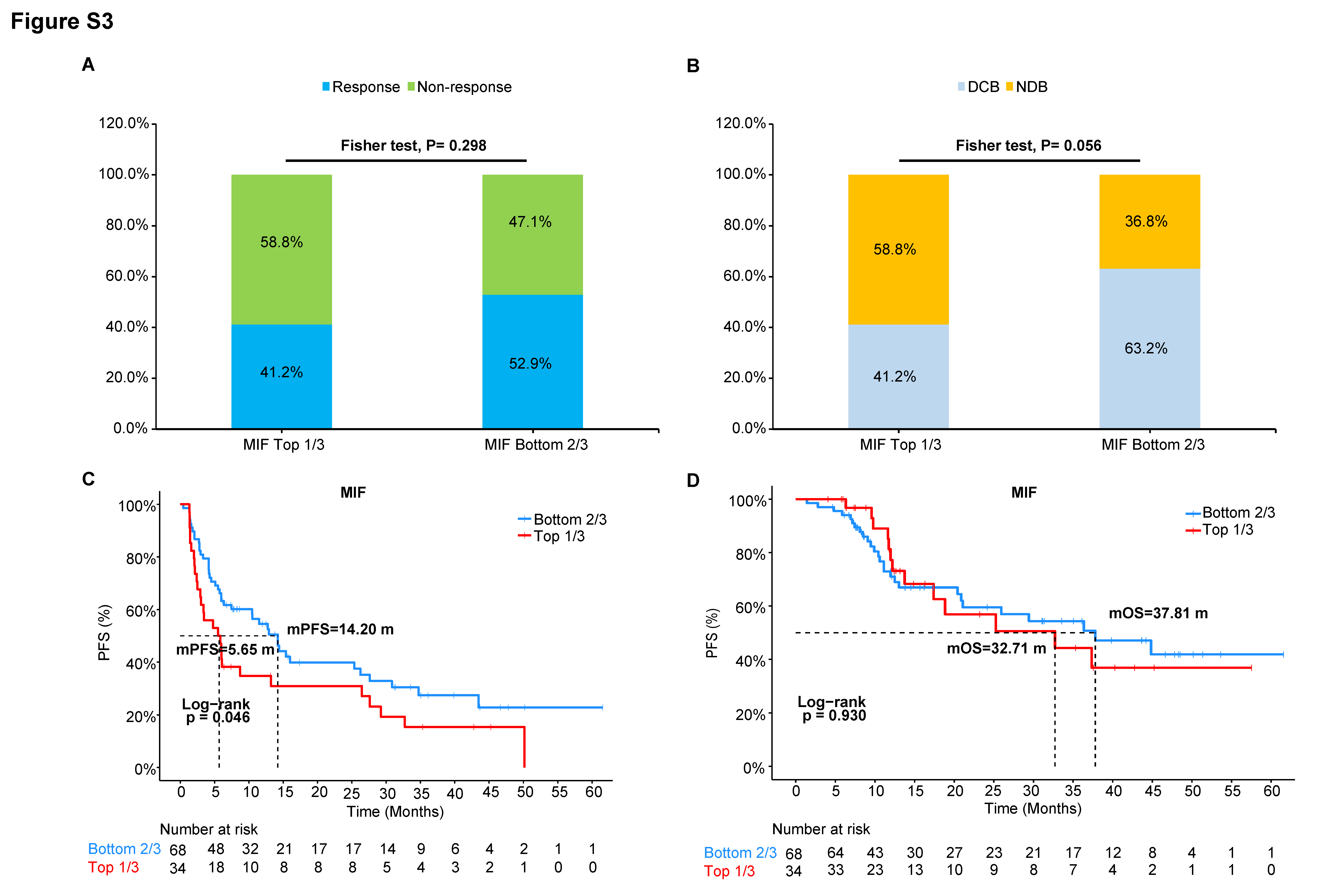

Supplement: Supplementary Figure 3 — The association of pretreatment circulating MIF with immunotherapy efficacy in NSCLC. (A) Response or nonresponse and (B) DCB or NDB of the patient proportions according to the baseline levels of circulating MIF, stratified by the cutoffs of the top 33% and bottom 67%. Kaplan-Meier curves of (C) PFS or (D) OS according to the circulating MIF level at baseline. DCB, durable clinical benefit. NDB, no durable benefit. [file Image_3.tif]

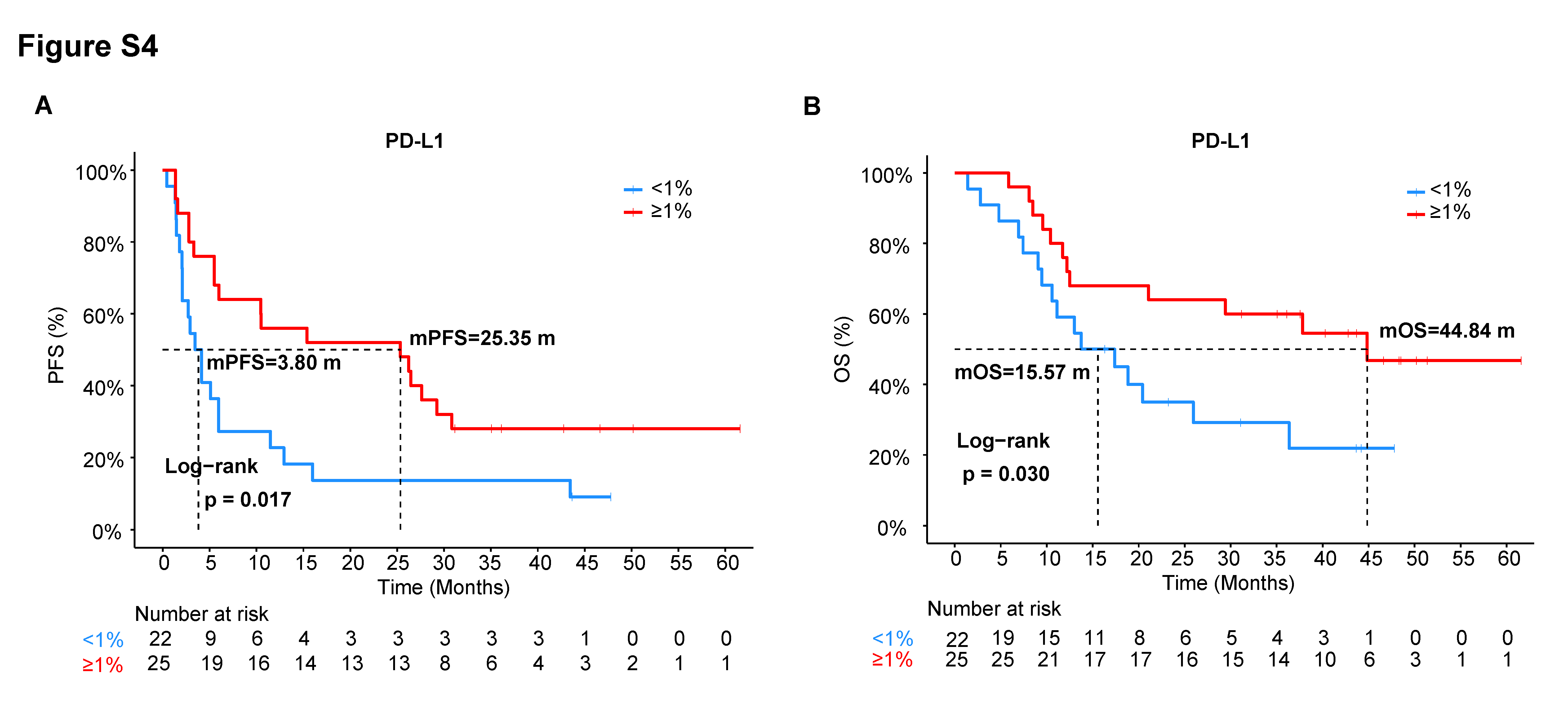

Supplement: Supplementary Figure 4 — The association of PD-L1 status with survival in patients who received mono-immunotherapy. Kaplan-Meier curves of (A) PFS and (B) OS according to the pretreatment PD-L1 expression status. [file Image_4.tif]

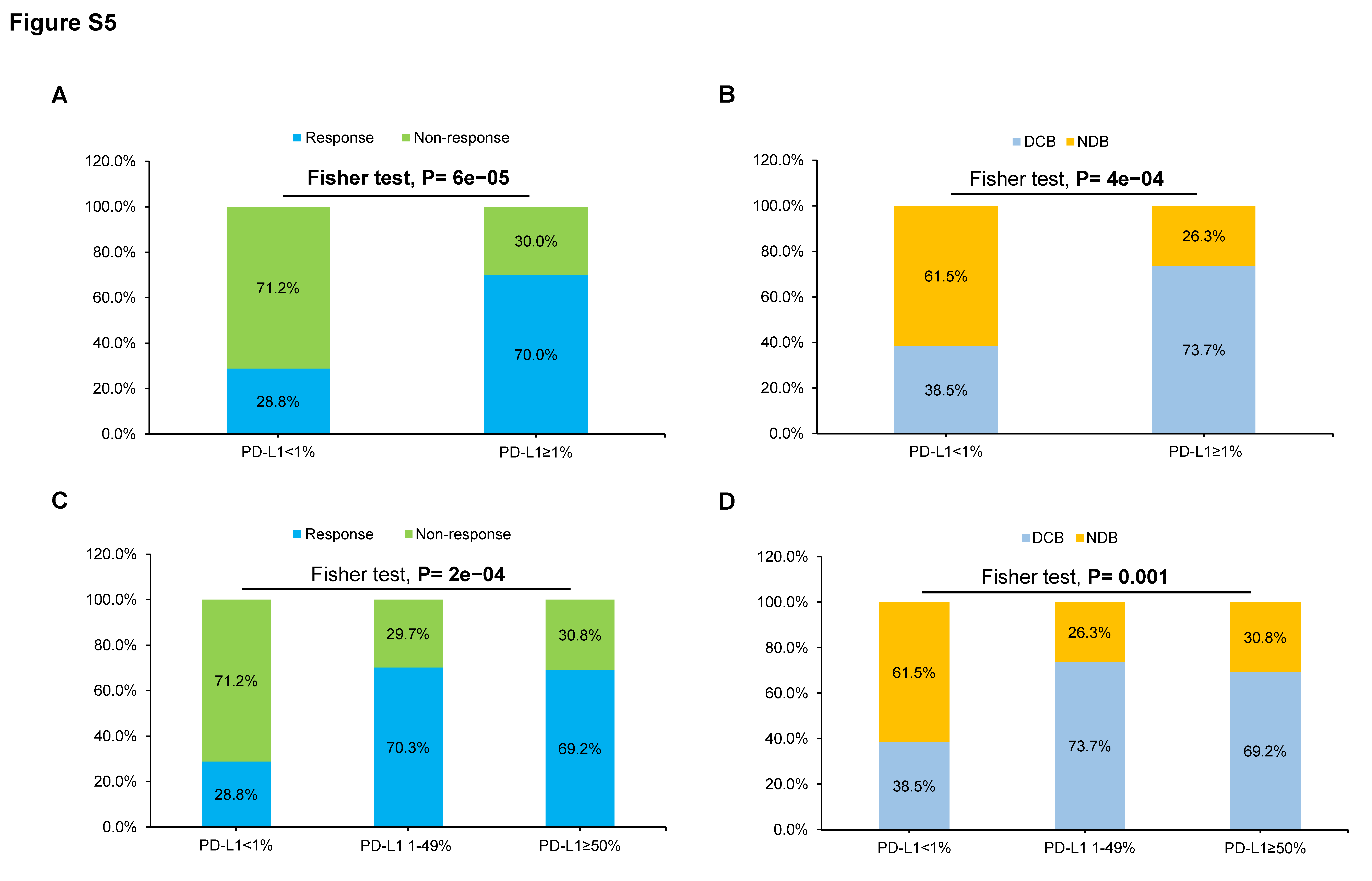

Supplement: Supplementary Figure 5 — The association of PD-L1 expression with immunotherapy response. (A) Response or nonresponse and (B) DCB or NDB of the patient proportions according to the PD-L1 expression levels (TPS≥1% and <1%). (C) Response or nonresponse and (D) DCB or NDB of the patient proportions stratified by PD-L1 status (TPS<1%, 1-49%, and ≥50%). TPS, tumor proportion score; DCB, durable clinical benefit; NDB, no durable benefit. [file Image_5.tif]

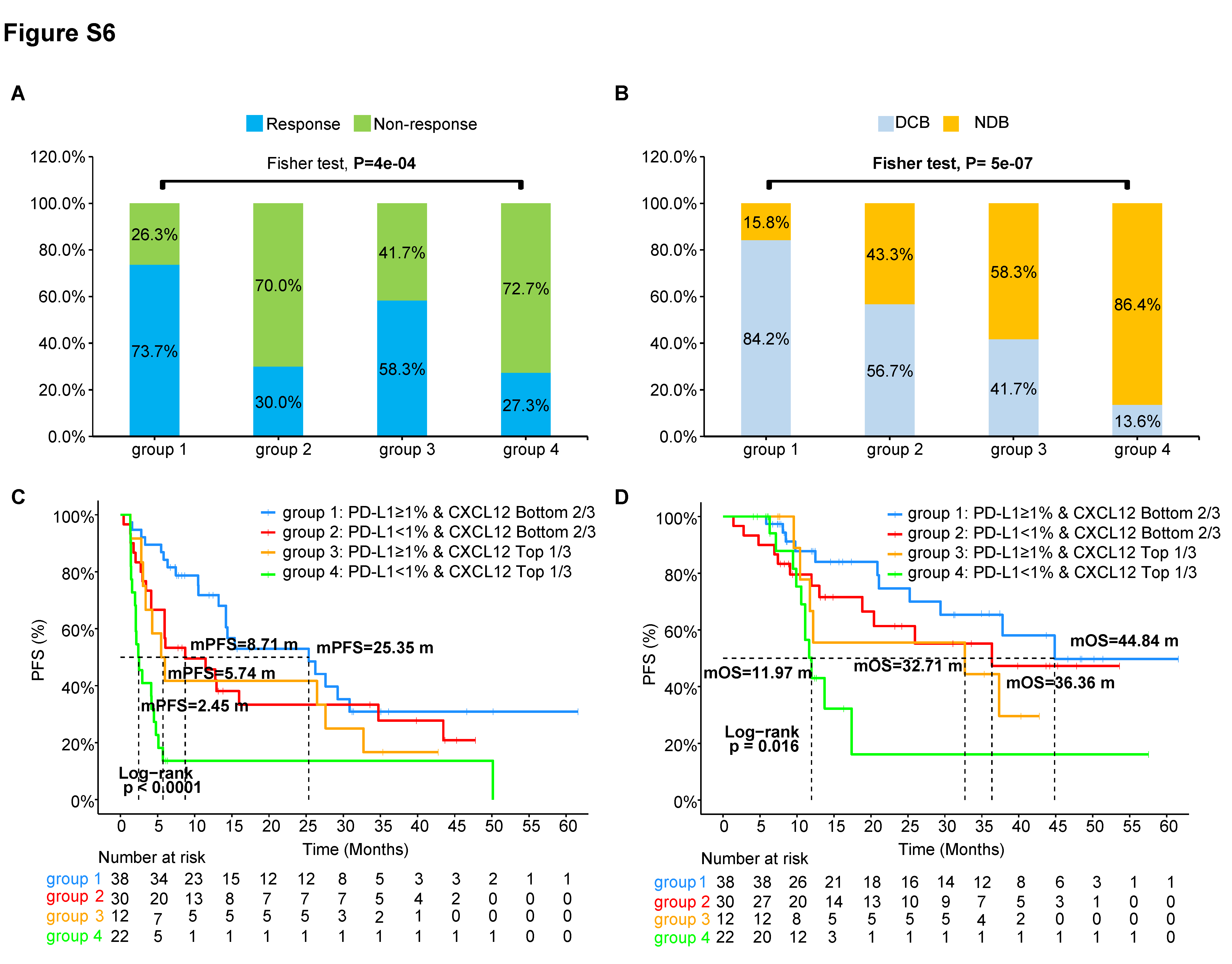

Supplement: Supplementary Figure 6 — Pretreatment PD-L1 expression plus circulating CXCL12 levels improve the prediction performance of immunotherapy outcomes. (A) Response or nonresponse and (B) DCB or NDB of the patient proportions in four groups stratified by the PD-L1 TPS plus CXCL12 level at baseline. Kaplan-Meier analysis of (C) PFS or (D) OS stratified by the PD-L1 expression plus CXCL12 level at baseline. The patients were divided into four groups: group 1 (TPS≥1% and CXCL12 bottom 67%); group 2 (TPS<1% and CXCL12 bottom 67%); group 3 (PD-L1≥1% and CXCL12 top 33%); and group 4 (PD-L1 < 1% and CXCL12 top 33%). TPS, tumor proportion score; DCB, durable clinical benefit; NDB, no durable benefit. [file Image_6.tif]
