# Supplementary material for: Serum cytokine analysis in a cohort of advanced non-small cell lung cancer treated with PD-1 inhibitors reveals predictive markers of CXCL12
Source: Front Immunol. 2023 Jun 9;14:1194123. doi: 10.3389/fimmu.2023.1194123 (PMC10288851; doi:10.3389/fimmu.2023.1194123)
Supplement: Supplementary file 7 [file Table_1.docx]

Table S1. List of cytokines studied in this study.

| CCL1 | CXCL10 | IFN-γ | IL-17E (IL-25) | IL-21 | IL-8 |
| --- | --- | --- | --- | --- | --- |
| CCL2 | CXCL11 | IL-10 | IL-18 | IL-27 | MIF |
| CCL5 | CXCL12 | IL-12 | IL-1A | IL-32A | MIP-1α |
| MIP-1β | CD40Ligand | G-CSF | IL-13 | IL-1B | IL-4 |
| serpinE1 | Complement C5 | GM-CSF | IL-16 | IL-1RA | IL-5 |
| TNF-a | CXCL1 | ICAM-1 | IL-17A | IL-2 | IL-6 |
| TREM-1 |  |  |  |  |  |
