# Supplementary material for: Serum cytokine analysis in a cohort of advanced non-small cell lung cancer treated with PD-1 inhibitors reveals predictive markers of CXCL12
Source: Front Immunol. 2023 Jun 9;14:1194123. doi: 10.3389/fimmu.2023.1194123 (PMC10288851; doi:10.3389/fimmu.2023.1194123)
Supplement: Supplementary file 8 [file Table_2.doc]

|  | **Cohort**  **N = 102** | **PD-L1 < 1%**  **n = 52** | **PD-L1 1–49%**  **n = 37** | **PD-L1 ≥ 50%**  **n = 13** |
| --- | --- | --- | --- | --- |
| **mPFS**  **(95% CI), m** | 10.49 (5.95-16.60) | 4.64 (2.96-11.47) | 25.35 (10.49-NR) | 27.62 (5.82-NR) |
| **mOS**  **(95%CI), m** | 36.36 (21.07–NR) | 20.42 (13.02–NR) | 37.81 (29.42–NR) | NR (21.07–NR) |
| **DCB, n (%)** | 57 (55.9%) | 20 (38.5%) | 28 (73.7%) | 9 (69.2%) |
| **ORR, n (%)** | 50 (49.0%) | 15 (28.8%) | 26 (70.3%) | 9 (69.2%) |
| **SD, n (%)** | 33 (32.4%) | 21 (40.4%) | 8 (21.6%) | 4 (30.8%) |
| **PD, n (%)** | 19 (18.6%) | 16 (30.8%) | 3 (8.1%) | 0 (0%) |
| **DCR, n (%)** | 83 (81.4%) | 35 (69.2%) | 34 (91.9%) | 13 (100.0%) |

**Table S2: ORR, PFS, and OS by PD-L1 status in NSCLC patients received immunotherapy.**

mPFS, median progression-free survival; mOS, median overall survival; NR, not reached; CI, confidence interval; DCB, durable clinical benefit; ORR, objective response rate; SD, stable disease; PD, progressive disease; DCR, disease control rate.
