# Supplementary material for: Serum cytokine analysis in a cohort of advanced non-small cell lung cancer treated with PD-1 inhibitors reveals predictive markers of CXCL12
Source: Front Immunol. 2023 Jun 9;14:1194123. doi: 10.3389/fimmu.2023.1194123 (PMC10288851; doi:10.3389/fimmu.2023.1194123)
Supplement: Supplementary file 9 [file Table_3.doc]

**Table S3: Univariate Cox analysis of PFS or OS by clinical parameters.**

|  | **PFS** | | **OS** | |
| --- | --- | --- | --- | --- |
| **Factor** | **p value** | **HR (95% CI)** | **p value** | **HR (95% CI)** |
| Sex  Male vs. Female | 0.336 | 0.74 (0.39-1.42) | 0.936 | 1.04 (0.41-2.66) |
| Age   ≥65 y vs. <65 y | 0.686 | 0.90 (0.55-1.49) | 0.787 | 1.09 (0.57-2.10) |
| Smoking_status  Yes vs. No | 0.751 | 0.91 (0.49-1.67) | 0.825 | 1.10 (0.46-2.63) |
| Pathology  SCC vs. ADC | 0.884 | 0.97 (0.60-1.54) | 0.844 | 1.06 (0.57-1.99) |
| Brain Metastasis   Yes vs. No | 0.661 | 0.82 (0.33-2.03) | 0.363 | 1.54 (0.60-3.95) |
| Liver Metastasis   Yes vs. No | 0.098 | 1.71 (0.90-3.27) | **0.012** | 2.52 (1.20-5.31) |
| irAEs  Yes vs. No | **0.003** | 0.44 (0.25-0.76) | **0.018** | 0.37 (0.15-0.87) |
| Line of treatment  2 vs. 1 | 0.154 | 1.41 (0.88-2.25) | 0.125 | 1.68 (0.86-3.29) |
| Treatment strategy  Mono-immunotherapy vs. Chemo-immunotherapy | 0.324 | 0.79 (0.49-1.27) | 0.132 | 1.69 (0.85-3.35) |

irAE, immune-related adverse events; TPS, tumor proportion score; CI, confidence interval; HR, hazard ratio.
