# Supplementary material for: Serum cytokine analysis in a cohort of advanced non-small cell lung cancer treated with PD-1 inhibitors reveals predictive markers of CXCL12
Source: Front Immunol. 2023 Jun 9;14:1194123. doi: 10.3389/fimmu.2023.1194123 (PMC10288851; doi:10.3389/fimmu.2023.1194123)
Supplement: Supplementary file 10 [file Table_4.docx]

|  | **Mono-immunotherapy (n=47)** | | | | **Chemo-immunotherapy (n=55)** | | | |
| --- | --- | --- | --- | --- | --- | --- | --- | --- |
|  | **PFS** | | **OS** | | **PFS** | | **OS** | |
| **Factor** | **HR (95% CI)** | **p value** | **HR (95% CI)** | **p value** | **HR (95% CI)** | **p value** | **HR (95% CI)** | **p value** |
| PD_L1 TPS  ≥1% vs. <1% | 0.46 (0.24-0.88) | **0.017** | 0.44 (0.20-0.94) | **0.030** | 0.52 (0.25-1.08) | 0.075 | 0.91 (0.29-2.92) | 0.876 |
| Liver Metastasis   Yes vs. No | 1.73 (0.76-3.93) | 0.189 | 2.14 (0.91~5.05) | 0.074 | 1.64 (0.57-4.74) | 0.358 | 3.86 (0.79-18.90) | 0.073 |
| irAEs   Yes vs. No | 0.53 (0.24-1.16) | 0.105 | 0.29 (0.00-0.96) | **0.031** | 0.35 (0.15-0.82) | **0.011** | 0.61 (0.16-2.27) | 0.457 |
| CXCL12  Top 1/3 vs. Bottom 2/3 | 1.46 (0.74-2.86) | 0.272 | 1.68(0.77-3.68) | 0.186 | 2.95 (1.45-6.01) | **0.002** | 3.72 (1.17-11.80) | **0.017** |
| MIF  Top 1/3 vs. Bottom 2/3 | 0.9 9(0.51-1.94) | 0.973 | 0.67 (0.31-1.59) | 0.390 | 1.70 (0.83-3.46) | 0.143 | 1.81 (0.56-5.84) | 0.316 |

**Table S4: Univariate analyses of clinical parameters, PD-L1 status and CXCL12 level in patients received mono-immunotherapy or chemo-immunotherapy combination therapy.**

irAE, immune-related adverse events; TPS, tumor proportion score; CI, confidence interval; HR, hazard ratio.
